# Regulatory T Cell in Kidney Transplant: The Future of Cell Therapy?

**DOI:** 10.3390/antib14020049

**Published:** 2025-06-17

**Authors:** Ahmad Matarneh, Meet Patel, Kinna Parikh, Amanda Karasinski, Gurwant Kaur, Vaqar Shah, Nasrollah Ghahramani, Naman Trivedi

**Affiliations:** 1 Division of Nephrology, Department of Medicine, Penn State Health Milton S. Hershey Medical Center, Hershey, PA 17033, USA; akarasinski@pennstatehealth.psu.edu (A.K.); gkaur1@pennstatehealth.psu.edu (G.K.); vshah3@pennstatehealth.psu.edu (V.S.); nghahramani@pennstatehealth.psu.edu (N.G.); ntrivedi1@pennstatehealth.psu.edu (N.T.); 2 Department of Internal Medicine, Western Reserve Hospital, Cuyahoga Falls, OH 44223, USA; meetpatel13@gmail.com (M.P.); parikhkinna9522@gmail.com (K.P.)

**Keywords:** kidney transplant, regulatory T cells, immunosuppressive therapy, treg adoptive cell therapy, graft versus host disease

## Abstract

The long-term use of immunosuppressive drugs following kidney transplantation increases the risk of life-threatening infections, malignancies, and, paradoxically, eventual allograft rejection. Therefore, achieving a balance between over-immunosuppression and under-immunosuppression is critical to optimizing patient outcomes. One promising approach is immune cell-based therapy using suppressor immune cells to modulate the immune response more precisely. Among these, regulatory T cells (Tregs) are the most extensively studied and have shown significant potential in the post-transplant setting. Tregs are broadly categorized into thymus-derived and peripherally derived subsets. Physiologically, they play key roles in maintaining immune tolerance, including in autoimmune diseases and within the tumor microenvironment. Their immunosuppressive functions are mediated through both contact-dependent and contact-independent mechanisms. Studies investigating the use of Tregs following kidney transplantation have shown encouraging results. This review summarizes the biology of Tregs and highlights current evidence supporting their role in transplant immunotherapy.

## 1. Introduction

Kidney transplantation remains the most effective treatment for patients with end-stage renal disease (ESRD), offering improved survival, quality of life, and reduced healthcare burden compared to long-term dialysis [1]. The success of transplantation, however, is contingent upon long-term immunosuppressive therapy to prevent graft rejection. Standard regimens typically include calcineurin inhibitors (CNIs) such as tacrolimus and cyclosporine, antiproliferative agents like mycophenolate mofetil, and glucocorticoids. These drugs have significantly reduced the incidence of acute rejection and improved early graft survival [2].

However, these pharmacologic agents are not without limitations. CNIs, particularly tacrolimus, the cornerstone of maintenance immunosuppression, are associated with both acute and chronic nephrotoxicity, leading to arteriolar injury, tubular atrophy, and interstitial fibrosis [3]. Additionally, CNIs can impair endothelial function, promote thrombotic microangiopathy (TMA), and contribute to progressive chronic allograft dysfunction. Beyond renal injury, they increase the risk of metabolic complications (e.g., post-transplant diabetes mellitus), neurotoxicity, and opportunistic infections, including BK virus nephropathy (BKVN). Long-term immunosuppression also suppresses tumor surveillance mechanisms, elevating the risk of de novo malignancies, such as lymphoproliferative disorders and solid tumors [4].

Despite advances in immunosuppressive strategies, chronic rejection remains a major cause of late allograft loss, often driven by subclinical immune activation that escapes pharmacologic control. Histologically, chronic rejection includes chronic active antibody-mediated rejection (ABMR) and T cell-mediated rejection (TCMR), both of which involve progressive inflammation, vascular remodeling, and fibrosis [5]. Moreover, the delicate balance between under- and over-immunosuppression is difficult to maintain and is highly individualized, often requiring frequent monitoring and drug adjustments.

Globally, over 100,000 kidney transplants are performed each year, with the United States, China, Brazil, and India leading in transplant volumes [6,7]. However, this represents a small fraction of the global ESRD population, which exceeds 3 million individuals on renal replacement therapy [8]. In the United States alone, more than 90,000 patients await a kidney transplant, with a median wait time of 3–5 years [9]. This unmet demand underscores the need for innovations, like Treg therapy, that may improve long-term graft survival.

Given these challenges, there has been growing interest in precision immunomodulation, aiming to promote immune tolerance while minimizing systemic immunosuppression. Among the most promising candidates for this purpose are regulatory T cells (Tregs), a naturally occurring subset of CD4^+^ T cells that maintain peripheral immune tolerance and prevent autoimmunity [10]. Tregs are defined by their expression of CD25, low CD127, and the master transcription factor FOXP3, which is critical for their development and suppressive function [11].

Tregs mediate immune regulation via both contact-dependent (e.g., CTLA-4, Fas–FasL, LAG-3) and contact-independent mechanisms (e.g., IL-10, TGF-β, IL-35 secretion), modulating the activity of effector T cells, B cells, dendritic cells, macrophages, and NK cells [12]. Importantly, their immunosuppressive effects are antigen-specific, offering the potential to prevent graft rejection without compromising global immune competence. Dysregulation of Tregs has been implicated in a variety of immunopathologic conditions, including autoimmune disease, transplant rejection, and even tumor escape in malignancy [13].

Recent preclinical and early-phase clinical trials have demonstrated the feasibility, safety, and early efficacy of adoptive Treg therapy in solid organ transplantation, including kidney transplants. Autologous ex vivo-expanded Tregs have shown promise in reducing alloimmune responses, delaying or minimizing calcineurin inhibitor use, and promoting long-term graft function. Moreover, advances in antigen-specific Treg generation and CAR-Treg engineering hold the potential to enhance the precision and durability of this approach [14].

Although Treg-based therapies are currently in early-phase clinical trials, accumulating evidence supports their safety and feasibility. However, translating these findings into routine clinical practice requires large-scale trials with longer follow-up, cost-effectiveness evaluations, and integration into existing transplant care models.

In this review, we present an in-depth exploration of the biology, classification, and immunosuppressive mechanisms of regulatory T cells, followed by a comprehensive discussion of clinical applications, trial data, and challenges associated with Treg-based immunotherapy in kidney transplantation. As we transition toward more tailored and biologic-based approaches, Treg therapy represents a paradigm shift with the potential to improve long-term outcomes and reduce the toxic burden of conventional immunosuppressive drugs.

## 2. Types of Tregs

**Figure 1:**
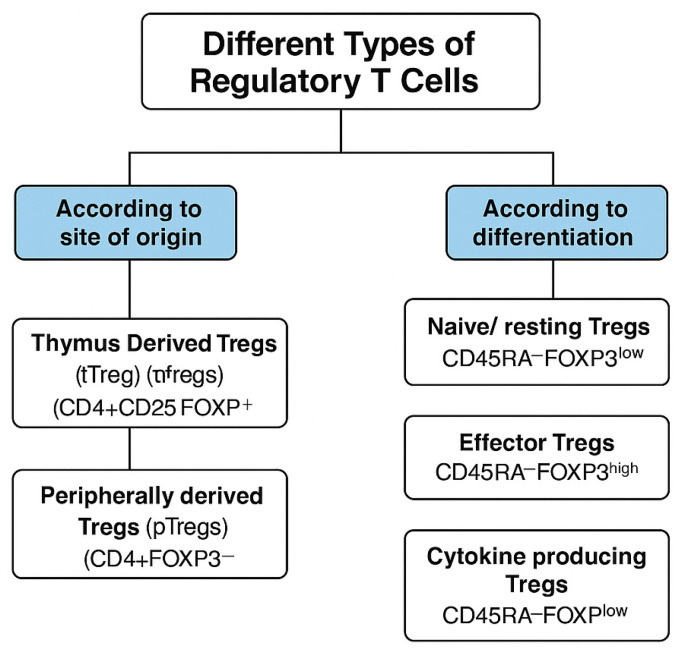
Types of Treg cells according to their site of origin and differentiation.

Regulatory T cells (Tregs) are a specialized subset of T cells that play a crucial role in maintaining immune tolerance and preventing excessive immune responses. They can be broadly classified based on their site of origin into thymus-derived Tregs (tTregs) and peripherally derived Tregs (pTregs) [12] (Figure 1).

Thymus-derived Tregs (tTregs), also known as natural Tregs (nTregs), develop in the thymus from CD4+ thymocytes and differentiate into CD4+ CD25+ FOXP3+ Tregs. These cells are predominantly found in the blood and lymph nodes and have high auto-affinity, meaning they recognize self-antigens with high specificity [15]. Their primary function is to provide tolerance against autoantigens, thereby preventing autoimmune responses.

In contrast, peripherally derived Tregs (pTregs) are generated outside the thymus from CD4+ FOXP3- conventional T cells in response to antigenic stimulation. Unlike tTregs, pTregs primarily recognize non-self-antigens, such as those present in the gut, airways, and commensal microbiota, playing a key role in suppressing local inflammation. Additionally, they are essential for maternal–fetal tolerance and preventing excessive immune reactions to commensal bacteria [16].

Tregs can also be generated artificially in vitro, known as induced Tregs (iTregs). These cells are produced in cell culture by stimulating CD4+ T cells with anti-CD3 in the presence of cytokines, like IL-10 and TGF-β. While iTregs share functional properties with natural Tregs, they are experimentally derived and serve as an important tool in immunological research [17].

Beyond their origin, Tregs can also be categorized based on their differentiation and activation status. Naïve or resting Tregs (CD45RA + FoxP3low) are the most stable subset of Tregs and have the potential to differentiate into more specialized Treg subsets [18]. Effector Tregs (CD45RA−FoxP3high) exhibit strong immunosuppressive activity and play a direct role in immune regulation. Another subset, cytokine-producing Tregs (CD45RA−FoxP3low), modulate immune responses primarily through the production of regulatory cytokines, such as IL-10 and TGF-β, rather than direct cell–cell interactions [19]

Importantly, tTregs are more phenotypically stable due to FOXP3 promoter demethylation, whereas pTregs and iTregs exhibit plasticity and are prone to converting into pro-inflammatory phenotypes in hostile cytokine environments [20]. This susceptibility to phenotypic drift may reduce their therapeutic utility in inflamed grafts, emphasizing the need to use stabilized or genetically modified Tregs for transplant purposes.

## 3. Comparison with Other Cell-Based Therapies

**Figure 2:**
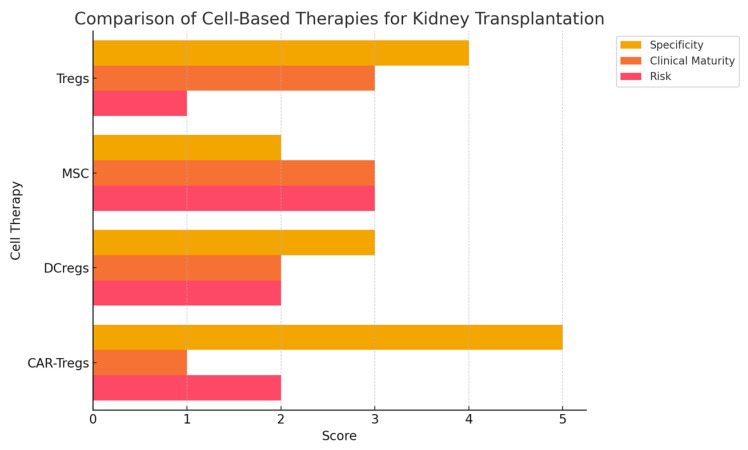
Comparison between different cell-based therapies.

Several immune-modulatory cell therapies have been investigated for transplant tolerance. Figure 2 shows a comparison between different cell-based therapies:

1. Regulatory Dendritic Cells (DCregs)—These cells induce antigen-specific tolerance by modulating T cell responses. Unlike Tregs, their immunosuppressive effects are indirect and may be less durable.
2. Mesenchymal Stromal Cells (MSCs)—MSCs exhibit anti-inflammatory properties and have been tested in renal transplantation, but concerns exist regarding their potential to differentiate into fibrogenic cells.
3. Transitional B Cells—These cells suppress immune responses via IL-10 production but are less studied compared to Tregs.

Among these, Tregs remain the most extensively characterized and have shown superior efficacy in preventing graft rejection [21].

## 4. Role of Tregs

Physiologically, Tregs accumulate in non-lymphoid tissues and barrier tissues, like skin, lungs and the GI tract. Once inflammation sets in, Tregs migrate to the local site and to the sentinel lymphoid tissue to dampen and curtail immune responses [22]. Reduced capacity of pTregs to suppress effector T cell proliferation and INF-*γ* production leads to development of autoimmune diseases, like multiple sclerosis, Type I DM, myasthenia gravis, and psoriasis. Also, loss of function gene mutation is associated with IPEX (immunodysregulation polyendocrinopathy enteropathy X linked syndrome). Additionally, Tregs play a crucial role in the tumor microenvironment. Animal tumor tissues show high infiltration by Tregs [23]. Studies on murine models have demonstrated that Tregs ablation produce a rapid immune response against tumor and improve chemotherapy efficacy.

## 5. Tregs Immunosuppressive Mechanisms

Tregs suppress the actions of a large number of immune cells, including B cells, CD4+ cells, cytotoxic CD8+ cells, NK cells, macrophages, dendritic cells and neutrophils. This is achieved by contact-dependent as well as contact-independent mechanisms.

Contact-dependent mechanisms include induction of apoptosis, restriction of the T effector cells activation, prevention of APC maturation, CD39/CD73-mediated inhibition of the nuclear factor of activated T cells (NFAT) and IL-2 transcription [24]. Apoptosis induction occurs in response to interaction of Cytotoxic T lymphocyte Antigen-4 (CTLA-4) with Programmed cell death receptor 1 (PD 1); Granzymes A and Granzyme B; through Fas/Fas ligand pathway, TRAIL (TNF-related apoptosis-inducing ligand), IL-2 deprivation or via Galectin-9/T cell immunoglobulin and mucin domain-3 (TIM-3) pathway [25]. Additionally, interaction of CTLA-4 with CD80/86 on the APCs induces a negative signal to restrict the activation of T effector cells. Also, binding of cell surface lymphocyte activation gene 3 (LAG3) to MHC II of the APCs prevents APC maturation and subsequently prevents activation of T effector cells. Another method of contact dependent immunosuppression includes CD39/CD73-mediated increased intracellular cAMP of Tregs transferred to T effector cells through gap junctions, which upregulates inducible cAMP early repressor (ICER). This leads to inhibition of the nuclear factor of activated T cells (NFAT) and IL-2 transcription [26]. These disruptions in the metabolic pathway through adenosine from ectoenzymes cause immunosuppression.

Contact-independent mechanisms comprise in vivo secretion of anti-inflammatory cytokines and transfer of microRNA. In vivo secretion of anti-inflammatory cytokines, like IL-10, TGF-β and IL-35, from Tregs inhibits T cell activation, whereas transfer of microRNA (miRNA) from Tregs through exosomes silences the T effector cells, which ultimately restricts further proliferation and production of proinflammatory cytokines [27].

In the context of transplantation, Tregs predominantly suppress alloimmune responses by downregulating APC co-stimulation (via CTLA-4) and secreting IL-10 and TGF-β. This differs from autoimmunity, where Tregs often suppress autoreactive T cells directly through IL-2 consumption or cytotoxicity. Inflammatory signals in allografts, such as IFN-γ or IL-6, may impair Treg stability, which necessitates the development of pro-tolerogenic strategies for clinical use [28].

## 6. Current Studies

### 6.1. Early Clinical Trials: Safety and Feasibility of Treg Therapy

The initial studies evaluating the safety and feasibility of Treg therapy in kidney transplantation laid the groundwork for its clinical application. Early-phase trials primarily focused on autologous Treg expansion, wherein patient-derived CD4+ CD25+ FOXP3+ cells were isolated, expanded ex vivo, and reinfused to modulate immune responses. These studies demonstrated that Treg infusion was well tolerated, with no immediate adverse effects, such as cytokine-release syndrome or exacerbation of opportunistic infections. Furthermore, no significant increase in graft rejection was observed following Treg administration [29].

One of the pivotal first-in-human trials demonstrated that Treg adoptive transfer in solid organ transplantation was feasible and safe. The study enrolled a small cohort of renal transplant recipients and administered ex vivo-expanded polyclonal Tregs at various doses. The findings indicated that infused Tregs persisted in circulation for a prolonged duration and retained their immunosuppressive function. However, variability in patient responses suggested that additional factors, such as baseline immune status, pre-existing sensitization, and donor-specific antigen load, could influence the effectiveness of therapy [30].

A notable distinction between Treg therapy in kidney versus liver transplantation has emerged from comparative clinical studies. A study in 2016 reported promising outcomes in liver transplant recipients, where 7 of 10 patients achieved complete withdrawal of immunosuppressive therapy following ex vivo-expanded Treg infusion [31]. In contrast, Koyama et al. [31] conducted a pioneering Phase I study from 2009 to 2012 involving 16 kidney transplant recipients using the same Treg protocol. Unfortunately, acute rejection occurred in seven patients, and none achieved complete immunosuppression withdrawal; all required maintenance on reduced immunosuppressive regimens. These findings underscore the organ-specific challenges of inducing tolerance in kidney transplantation and highlight the heightened alloimmune reactivity compared to liver allografts.

### 6.2. Phase I/II Trials: Immunomodulation and Reduction in Immunosuppressive Burden

Subsequent Phase I/II trials aimed to assess the potential of Tregs in reducing dependence on conventional immunosuppressive drugs while maintaining graft survival. One key study investigated the impact of autologous Treg infusion in kidney transplant patients receiving standard immunosuppressive therapy. The results revealed that patients receiving Treg therapy exhibited lower rates of acute cellular rejection (ACR) and demonstrated a gradual tapering of immunosuppressive drugs, particularly calcineurin inhibitors (CNIs). Notably, a significant reduction in donor-specific antibody (DSA) formation was observed, suggesting that Tregs contributed to attenuating alloimmune responses while preserving immune homeostasis [32].

Another trial utilized antigen-specific Tregs expanded against donor-derived alloantigens, rather than polyclonally expanded Tregs. This study hypothesized that antigen-specific Tregs would provide more precise immunomodulation, thereby reducing the risk of generalized immunosuppression and associated complications. Findings from this trial demonstrated improved graft function and a decrease in pro-inflammatory cytokine profiles in treated patients. However, despite these promising results, concerns remained regarding the stability of ex vivo-expanded Tregs over time and their ability to maintain suppressive function in vivo [33].

Despite these promising findings, limitations remain. Many trials enrolled small, highly selected populations, with variable Treg expansion protocols and short follow-up durations. Moreover, the immunologic environment in humans is more heterogeneous than in preclinical models, potentially affecting reproducibility. As such, caution is warranted in extrapolating early-phase outcomes to broader clinical settings [34].

A notable study conducted in Europe involved the infusion of Tregs in renal transplant recipients undergoing tapering of standard immunosuppression. The trial showed that patients who received Treg therapy maintained stable renal function with a lower incidence of chronic allograft dysfunction. Additionally, immune profiling revealed a shift towards a regulatory immune environment, characterized by increased IL-10 and TGF-β secretion, reduction in Th1/Th17 pro-inflammatory responses, and an expansion of regulatory B cells (Bregs), suggesting a systemic immune tolerance induction [35]. Below is a summary of the main trials (Table 1).

### 6.3. Recent Advances: Antigen-Specific and Genetically Engineered Tregs

Recent trials have advanced beyond polyclonal Treg infusion by exploring the potential of antigen-specific Tregs and genetically engineered Tregs. Antigen-specific Tregs, generated using donor-derived antigen-presenting cells (APCs), have demonstrated superior immunoregulatory capacity compared to polyclonal Tregs. These studies suggest that antigen-specific Tregs selectively suppress alloimmune responses against the transplanted kidney without broadly suppressing the immune system, thereby reducing the risk of infections and malignancies [40].

Chimeric antigen receptor Tregs (CAR-Tregs) represent another groundbreaking advancement in this field. CAR-Tregs are genetically modified to express a chimeric receptor that recognizes donor-specific antigens with high affinity, allowing them to localize more efficiently to the graft and exert precise immunosuppressive effects [41]. Preclinical studies have shown that CAR-Tregs enhance long-term graft tolerance and reduce chronic rejection markers. Ongoing Phase I trials are evaluating the safety and efficacy of CAR-Tregs in human renal transplantation. If successful, this approach may offer a paradigm shift in transplant immunotherapy by providing targeted immune regulation while minimizing systemic immunosuppression [36].

### 6.4. Meta-Analysis and Systematic Reviews: Treg Therapy in Transplantation

Several meta-analyses have synthesized data from existing clinical trials to evaluate the overall impact of Treg therapy in kidney transplantation. A large meta-analysis comparing Treg therapy with standard immunosuppressive regimens reported that Treg-treated patients exhibited a significantly lower incidence of acute rejection, reduced de novo DSA formation, and decreased reliance on CNIs. Furthermore, kidney allograft survival rates were comparable or superior to those achieved with conventional immunosuppression, highlighting the therapeutic potential of Tregs [42].

Another systematic review assessed Treg therapy across different organ transplantation settings, including liver and heart transplantation, to identify commonalities and challenges. The review emphasized that while Treg therapy demonstrated promising immunomodulatory effects, variability in patient selection, dosing strategies, and ex vivo expansion protocols influenced outcomes. Notably, many early studies included multiple organ types (e.g., liver, heart, kidney), making it difficult to interpret kidney-specific effects. For example, the Koyama et al. [31] trial involved liver transplant patients, whereas Lai et al. [37] focused on kidney recipients. Future analyses should stratify outcomes by organ type to assess therapeutic efficacy more precisely in renal transplantation. Standardizing these parameters was deemed essential for the broader clinical application of Tregs [43].

### 6.5. Future Challenges and Research Directions

Despite encouraging results from clinical trials, one of the primary challenges in Treg therapy remains the optimization of ex vivo expansion protocols. Large-scale production of Tregs requires specialized cell culture facilities, cytokine supplementation, and stringent quality control measures to ensure stability and functionality. Current efforts focus on refining good manufacturing practice (GMP)-compliant protocols for large-scale expansion while preserving Treg lineage fidelity [44].

A major concern in Treg therapy is the long-term stability and persistence of infused cells. Studies have shown that some expanded Tregs may lose FOXP3 expression over time, leading to phenotypic drift and loss of suppressive function. To address this, researchers are investigating strategies, such as epigenetic modulation to enhance FOXP3 stability and prevent Treg plasticity [45].

Patient-specific factors, including genetic predisposition, prior sensitization, and immune system composition, influence the success of Treg therapy. Identifying reliable biomarkers that predict Treg efficacy and patient response remains a crucial area of research. Biomarkers, such as FOXP3 methylation status, cytokine profiles (IL-10, TGF-β, IL-35), and Treg-to-effector T cell ratios, are being explored for their potential to guide patient selection and treatment monitoring [46].

Conventional immunosuppressants influence Treg viability and expansion. CNIs, especially tacrolimus, impair IL-2 signaling crucial for Treg maintenance [11]. Conversely, mTOR inhibitors, like sirolimus, support Treg proliferation and may act synergistically with Treg-based therapies [47]. Tailoring immunosuppressive regimens to be Treg-compatible may enhance treatment outcomes and promote tolerance.

The cost of Treg therapy remains a significant barrier to widespread implementation. The need for specialized infrastructure, personalized cell expansion, and rigorous monitoring makes it an expensive therapeutic approach. Biologically, several hurdles remain. Patient heterogeneity—including immune history, sensitization status, and cytokine milieu—affects Treg function post-infusion. Furthermore, ex vivo-expanded Tregs may lose FOXP3 expression over time, increasing the risk of losing suppressive identity or converting into pathogenic effector T cells. Contamination with conventional T cells during expansion further complicates product purity. Advanced assays, such as FOXP3 methylation analysis and Treg:Tconv ratios, are being developed to ensure therapeutic consistency [48]. Efforts to develop cost-effective, off-the-shelf Treg products using universal donor cell lines are currently underway to enhance accessibility.

Although Tregs are known to suppress alloimmune responses, excessive Treg activity may lead to an increased risk of infections and malignancies. Striking the right balance between immune tolerance and immune surveillance is critical. Future studies will need to assess long-term immune competence in Treg-treated transplant recipients to mitigate the risk of opportunistic infections and post-transplant malignancies [49].

Regulatory agencies, such as the FDA and EMA, have yet to establish standardized guidelines for Treg-based therapies. The complexity of cell-based treatments necessitates rigorous clinical evaluation to ensure safety and efficacy. Additionally, ethical concerns regarding genetic modification of Tregs for enhanced function must be addressed before widespread clinical application [50].

A key challenge in Treg therapy is the lack of standardized methods for long-term monitoring. Unlike conventional drugs, there is no pharmacokinetic equivalent for infused Tregs [51]. Clinicians must rely on indirect markers, such as Treg frequencies in peripheral blood, cytokine panels, or methylation-based assays, to estimate persistence and function. Longitudinal immune profiling and functional testing may help define thresholds for reducing or stopping concomitant immunosuppression [52]. Establishing reliable markers of tolerance will be essential, not only for clinical safety but also for the broader acceptance of Treg-based protocols in transplant care. A significant barrier to effective long-term monitoring of Treg-based therapies is the lack of harmonized assays across institutions. Flow cytometry protocols, gating strategies, and Treg surface marker panels often vary, making cross-study comparisons difficult [53]. This variability limits the ability to define universal thresholds for tolerance or graft protection. The development of standardized immune monitoring frameworks, such as those proposed in The ONE Study and other multicenter collaborations, may allow for better comparability of results across trials [14]. These frameworks often include core marker panels (e.g., CD4^+^CD25^+^FOXP3^+^CD127^low), cytokine multiplexing, and epigenetic profiling of Treg stability and are essential for regulatory approval and clinical adoption.

Looking ahead, for Treg therapy to be widely adopted, several key milestones must be achieved. These include the identification of predictive biomarkers of response, development of off-the-shelf allogeneic Treg products, resolution of regulatory and ethical considerations, and cost-reduction strategies. The integration of Treg therapy into transplant care pathways will also require clinician education and standardized clinical protocols to ensure safe implementation.

To improve clinical utility, future studies should strive to quantify the extent and durability of immunosuppression reduction following Treg therapy. Currently, most reports remain qualitative, with limited numerical benchmarks for clinicians. Specifically, key questions remain unanswered: (1) To what extent can CNIs and steroids be safely tapered? (2) What immune monitoring markers—such as donor-specific antibodies, Treg:Teff ratios, or cytokine signatures—can guide this tapering? (3) How long does tolerance persist post-infusion? In the Koyama et al. [31] trial, despite Treg therapy, none of the 16 kidney transplant recipients could completely discontinue immunosuppressive drugs, while some studies report partial dose reductions with stabilization of graft function. Systematic quantification of these clinical outcomes is critical for translating Treg therapy from investigational to standard-of-care.

### 6.6. Antigen-Specific and Engineered Tregs: The Next Frontier

While polyclonal Tregs have demonstrated safety in early-phase trials, their broad specificity may limit therapeutic efficacy. Antigen-specific Tregs, enriched from alloantigen-stimulated cultures or generated through genetic engineering, offer a more targeted approach. These cells suppress immune responses more efficiently at sites of inflammation and alloantigen presentation, minimizing systemic immunosuppression. Engineered Tregs expressing antigen-specific T cell receptors (TCRs) or chimeric antigen receptors (CARs) represent a particularly promising advancement [54]. Preclinical studies have shown that CAR-Tregs specific for donor HLA molecules can localize to the graft and mediate localized immune regulation with enhanced potency compared to polyclonal Tregs [37]. These engineered Tregs can also be modified to improve stability, resistance to inflammatory cytokines, and trafficking to lymphoid or graft tissues. Despite their promise, several challenges remain. Manufacturing is technically demanding and costly, and safety concerns related to insertional mutagenesis and off-target effects must be addressed in clinical protocols [55]. Nevertheless, the refinement of antigen-specific Treg therapies may pave the way for precision tolerance strategies in transplantation.

### 6.7. Economic and Logistical Considerations

Despite its promise, Treg therapy remains expensive and technically demanding. Generating GMP-grade Tregs involves leukapheresis, ex vivo expansion, and quality control testing, all of which add significant costs. Estimates suggest that each dose can range between USD 30,000 and USD 50,000, which presents a barrier to routine use [56]. While these costs may be offset by reduced complications from long-term immunosuppression, this remains theoretical without long-term economic modeling. Advancements in cell expansion technology, use of third-party or allogeneic Tregs, and centralized manufacturing facilities could help lower the cost and increase access. Until then, affordability and resource availability will remain limiting factors in clinical adoption. For context, the total cost of a kidney transplant in the United States—including the procedure, initial hospitalization, and first-year immunosuppressive medications—can exceed USD 400,000. Annual maintenance immunosuppression can add USD 10,000–USD 15,000 more per year [57]. If Treg therapy can reduce rejection episodes and long-term immunosuppressive needs, it may be cost-effective in select patient populations, despite its upfront costs.

## 7. Conclusions

Treg therapy holds significant promise in kidney transplantation by reducing reliance on long-term immunosuppressive drugs and promoting immune tolerance. Although short-term outcomes have improved with current therapies, long-term graft survival remains limited by chronic rejection and drug-related toxicity. Tregs offer a potential solution, but clinical adoption requires addressing challenges related to stability, patient variability, and cost. Emerging advances in antigen-specific and genetically engineered Tregs may enhance the precision and durability of this approach, positioning Treg therapy as a future cornerstone of transplant immunotherapy.

## Figures and Tables

**Table 1:** Summary of key clinical trials on regulatory T Cell therapy in transplantation.

Study	Phase	Treg Type	Key Outcome
The ONE Study (2020) [36]	I/II	Polyclonal	Safe, reduced rejection
Lai et al. (2018) [37]	I	Ex vivo-expanded	Stable graft function
Bluestone et al. (2015) [38]	Pilot	Polyclonal	Well tolerated
Koyama et al. (2016) [31]	I/II	Ex vivo-expanded	Tolerance achieved in liver Tx
Trzonkowski et al. (2009) [39]	I	Polyclonal	Safe in GVHD; foundational
CAR-Treg (ongoing)	I	Genetically engineered	Awaiting results

## Data Availability

Not applicable. No new data were generated or analyzed in support of this article.

## References

[1] United States Renal Data System (2023). 2023 USRDS Annual Data Report: Epidemiology of Kidney Disease in the United States.

[2] Hansen C.M., Bachmann S., Su M., Budde K., Choi M. (2025). Calcineurin Inhibitor Associated Nephrotoxicity in Kidney Transplantation—A Transplant Nephrologist’s Perspective. Acta Physiol..

[3] Puri P., Bansal N. (2023). Renal Dysfunction After Liver Transplant: Is CNI Nephrotoxicity Overrated. J. Clin. Exp. Hepatol..

[4] Rubinstein J., Toner K., Gross T., Wistinghausen B. (2023). Diagnosis and management of post-transplant lymphoproliferative disease following solid organ transplantation in children, adolescents, and young adults. Best Pract. Res. Clin. Haematol..

[5] Tamargo C.L., Kant S. (2023). Pathophysiology of rejection in kidney transplantation. J. Clin. Med..

[6] Cron D.C., Husain S.A., King K.L., Mohan S., Adler J.T. (2023). Increased volume of organ offers and decreased efficiency of kidney placement under circle-based kidney allocation. Am. J. Transplant..

[7] Wang J.H., Hart A. (2021). Global perspective on kidney transplantation: United States. Kidney360.

[8] Liyanage T., Ninomiya T., Jha V., Neal B., Patrice H.M., Okpechi I., Zhao M.H., Lv J., Garg A.X., Knight J. (2015). Worldwide access to treatment for end-stage kidney disease: A systematic review. Lancet.

[9] UCaldwell J.S., Cheng X.S., Chertow G.M., Goldhaber-Fiebert J.D. (2025). Kidney transplant wait times under waiting list expansion scenarios. JAMA Netw. Open.

[10] Cheung J., Zahorowska B., Suranyi M., Wong J.K., Diep J., Spicer S.T., Verma N.D., Hodgkinson S.J., Hall B.M. (2022). CD4+ CD25+ T regulatory cells in renal transplantation. Front. Immunol..

[11] Vignali D.A., Collison L.W., Workman C.J. (2008). How regulatory T cells work. Nat. Rev. Immunol..

[12] Wing J.B., Tanaka A., Sakaguchi S. (2019). Human FOXP3+ regulatory T cell heterogeneity and function in autoimmunity and cancer. Immunity.

[13] Wegrzyn A.S., Kedzierska A.E., Obojski A. (2023). Identification and classification of distinct surface markers of T regulatory cells. Front. Immunol..

[14] Arjomandnejad M., Kopec A.L., Keeler A.M. (2022). CAR-T regulatory (CAR-Treg) cells: Engineering and applications. Biomedicines.

[15] Santamaria J.C., Borelli A., Irla M. (2021). Regulatory T cell heterogeneity in the thymus: Impact on their functional activities. Front. Immunol..

[16] Itahashi K., Irie T., Nishikawa H. (2022). Regulatory T-cell development in the tumor microenvironment. Eur. J. Immunol..

[17] Chen B.J., Zhao J.W., Zhang D.H., Zheng A.H., Wu G.Q. (2022). Immunotherapy of cancer by targeting regulatory T cells. Int. Immunopharmacol..

[18] Sakaguchi S., Miyara M., Costantino C.M., Hafler D.A. (2010). FOXP3+ regulatory T cells in the human immune system. Nat. Rev. Immunol..

[19] Floess S., Freyer J., Siewert C., Baron U., Olek S., Polansky J., Schlawe K., Chang H.D., Bopp T., Schmitt E. (2007). Epigenetic control of the foxp3 locus in regulatory T cells. PLoS Biol..

[20] McGovern J., Holler A., Thomas S., Stauss H.J. (2022). Forced Fox-P3 expression can improve the safety and antigen-specific function of engineered regulatory T cells. J. Autoimmun..

[21] Bashor C.J., Hilton I.B., Bandukwala H., Smith D.M., Veiseh O. (2022). Engineering the next generation of cell-based therapeutics. Nat. Rev. Drug Discov..

[22] Jiang Q., Yang G., Liu Q., Wang S., Cui D. (2021). Function and role of regulatory T cells in rheumatoid arthritis. Front. Immunol..

[23] Chen H., Zha J., Tang R., Chen G. (2023). T-cell immunoglobulin and mucin-domain containing-3 (TIM-3): Solving a key puzzle in autoimmune diseases. Int. Immunopharmacol..

[24] Grover P., Goel P.N., Greene M.I. (2021). Regulatory T cells: Regulation of identity and function. Front. Immunol..

[25] Okoye I.S., Coomes S.M., Pelly V.S., Czieso S., Papayannopoulos V., Wilson M.S. (2014). MicroRNA-containing T-regulatory-cell-derived exosomes suppress pathogenic T helper 1 cells. Immunity.

[26] Kaye J. (2023). Integrating T cell activation signals to regulate gene expression through cyclosporin-sensitive NFAT. J. Immunol..

[27] Brunstein C.G., Miller J.S., Cao Q., McKenna D.H., Hippen K.L., Curtsinger J., Defor T., Levine B.L., June C.H., Rubinstein P. (2011). Infusion of ex vivo expanded T regulatory cells in adults transplanted with umbilical cord blood: Safety profile and detection kinetics. Blood.

[28] West P.K., McCorkindale A.N., Guennewig B., Ashhurst T.M., Viengkhou B., Hayashida E., Jung S.R., Butovsky O., Campbell I.L., Hofer M.J. (2022). The cytokines interleukin-6 and interferon-α induce distinct microglia phenotypes. J. Neuroinflamm..

[29] Sánchez-Fueyo A., Whitehouse G., Grageda N., Cramp M.E., Lim T.Y., Romano M., Thirkell S., Lowe K., Fry L., Heward J. (2020). Applicability, safety, and biological activity of regulatory T cell therapy in liver transplantation. Am. J. Transplant..

[30] Oberholtzer N., Atkinson C., Nadig S.N. (2021). Adoptive transfer of regulatory immune cells in organ transplantation. Front. Immunol..

[31] Koyama I., Sakaguchi S., Todo S. (2020). Clinical efficacy of regulatory T-cell therapy in kidney transplantation: First-in-human phase I study. Transplantation.

[32] Mikami N., Sakaguchi S. (2023). Regulatory T cells in autoimmune kidney diseases and transplantation. Nat. Rev. Nephrol..

[33] Chen P.P., Cepika A.M., Agarwal-Hashmi R., Saini G., Uyeda M.J., Louis D.M., Cieniewicz B., Narula M., Amaya Hernandez L.C., Harre N. (2021). Alloantigen-specific type 1 regulatory T cells suppress through CTLA-4 and PD-1 pathways and persist long-term in patients. Sci. Transl. Med..

[34] Harden P.N., Game D.S., Sawitzki B., Van der Net J.B., Hester J., Bushell A., Issa F., Brook M.O., Alzhrani A., Schlickeiser S. (2021). Feasibility, long-term safety, and immune monitoring of regulatory T cell therapy in living donor kidney transplant recipients. Am. J. Transplant..

[35] Lu J., Li P., Du X., Liu Y., Zhang B., Qi F. (2021). Regulatory T cells induce transplant immune tolerance. Transpl. Immunol..

[36] Sawitzki B., Harden P.N., Reinke P., Moreau A., Hutchinson J.A., Game D.S., Tang Q., Guinan E.C., Battaglia M., Burlingham W.J. (2020). Regulatory cell therapy in kidney transplantation (The ONE Study): A harmonised design and analysis of seven non-randomised, single-arm, phase 1/2A trials. Lancet.

[37] Lai X., Zheng X., Mathew J.M., Gallon L., Leventhal J.R., Zhang Z.J. (2021). Tackling Chronic Kidney Transplant Rejection: Challenges and Promises. Front. Immunol..

[38] Bluestone J.A., Buckner J.H., Fitch M., Gitelman S.E., Gupta S., Hellerstein M.K., Herold K.C., Lares A., Lee M.R., Li K. (2015). Type 1 diabetes immunotherapy using polyclonal regulatory T cells. Sci. Transl. Med..

[39] Trzonkowski P., Bieniaszewska M., Juscinska J., Dobyszuk A., Krzystyniak A., Marek N., Myśliwska J., Hellmann A. (2009). First-in-man clinical trial of autologous CD4+CD25+CD127− regulatory T cells in patients with graft-versus-host disease and type 1 diabetes. Clin. Immunol..

[40] Mohseni Y.R., Tung S.L., Dudreuilh C., Lechler R.I., Fruhwirth G.O., Lombardi G. (2020). The future of regulatory T cell therapy: Promises and challenges of implementing CAR technology. Front. Immunol..

[41] Riet T., Chmielewski M. (2022). Regulatory CAR-T cells in autoimmune diseases: Progress and current challenges. Front. Immunol..

[42] Juneja T., Kazmi M., Mellace M., Saidi R.F. (2022). Utilization of Treg Cells in Solid Organ Transplantation. Front. Immunol..

[43] Hu M., Rogers N.M., Li J., Zhang G.Y., Wang Y.M., Shaw K., O’Connell P.J., Alexander S.I. (2021). Antigen Specific Regulatory T Cells in Kidney Transplantation and Other Tolerance Settings. Front. Immunol..

[44] Banas B., Krämer B.K., Krüger B., Kamar N., Undre N. (2020). Long-Term Kidney Transplant Outcomes: Role of Prolonged-Release Tacrolimus. Transplant. Proc..

[45] Mamlouk O., Nair R., Iyer S.P., Edwards A., Neelapu S.S., Steiner R.E., Adkins S.A., Hawkins M., Saini N., Devashish K. (2021). Safety of CAR T-cell therapy in kidney transplant recipients. Blood J. Am. Soc. Hematol..

[46] Jin B., Lu Z., Cheng C., Pei Y., Chen L., Yue Z., Lin A., Yang S., Mo Y., Jiang X. (2025). Factors associated with chronic calcineurin inhibitor nephrotoxicity in children with minimal-change disease. Ren. Fail..

[47] Yu J., Wei X., Gao J., Wang C., Wei W. (2023). Role of cyclosporin A in the treatment of kidney disease and nephrotoxicity. Toxicology.

[48] Liu Y. (2004). Epithelial to Mesenchymal Transition in Renal Fibrogenesis: Pathologic Significance, Molecular Mechanism, and Therapeutic Intervention. J. Am. Soc. Nephrol..

[49] Dikiy S., Rudensky A.Y. (2023). Principles of regulatory T cell function. Immunity.

[50] Shrestha B.M. (2017). Two Decades of Tacrolimus in Renal Transplant: Basic Science and Clinical Evidences. Exp. Clin. Transplant. Off. J. Middle East Soc. Organ Transplant..

[51] Hirsch H.H., Yakhontova K., Lu M., Manzetti J. (2016). BK Polyomavirus Replication in Renal Tubular Epithelial Cells Is Inhibited by Sirolimus, but Activated by Tacrolimus Through a Pathway Involving FKBP-12. Am. J. Transplant. Off. J. Am. Soc. Transplant. Am. Soc. Transpl. Surg..

[52] Shen C.L., Wu B.S., Lien T.J., Yang A.H., Yang C.Y. (2021). BK Polyomavirus Nephropathy in Kidney Transplantation: Balancing Rejection and Infection. Viruses.

[53] Ambalathingal G.R., Francis R.S., Smyth M.J., Smith C., Khanna R. (2017). BK Polyomavirus: Clinical Aspects, Immune Regulation, and Emerging Therapies. Clin. Microbiol. Rev..

[54] Jahan S., Scuderi C., Francis L., Neller M.A., Rehan S., Crooks P., Ambalathingal G.R., Smith C., Khanna R., John G.T. (2020). T-cell adoptive immunotherapy for BK nephropathy in renal transplantation. Transpl. Infect. Dis. Off. J. Transplant. Soc..

[55] Loupy A., Haas M., Roufosse C., Naesens M., Adam B., Afrouzian M., Akalin E., Alachkar N., Bagnasco S., Becker J.U. (2020). The Banff 2019 Kidney Meeting Report (I): Updates on and clarification of criteria for T cell- and antibody-mediated rejection. Am. J. Transplant. Off. J. Am. Soc. Transplant. Am. Soc. Transpl. Surg..

[56] Muckenhuber M., Wekerle T. (2023). T regulatory cell therapy: The price of specificity. Am. J. Transplant..

[57] Cheng X.S., Han J., Braggs-Gresham J.L., Held P.J., Busque S., Roberts J.P., Tan J.C., Scandling J.D., Chertow G.M., Dor A. (2022). Trends in cost attributable to kidney transplantation evaluation and waiting list management in the United States, 2012–2017. JAMA Netw. Open.

